# Developing Effective Cancer Vaccines Using Rendered-Inactive Tumor Cells

**DOI:** 10.3390/vaccines11081330

**Published:** 2023-08-05

**Authors:** Shushu Zhao, Shuting Wu, Sheng Jiang, Gan Zhao, Bin Wang

**Affiliations:** 1 Key Laboratory of Medical Molecular Virology, School of Basic Medical Sciences, Fudan University, Shanghai 200032, China; szhao@wistar.org (S.Z.); 19111010058@fudan.edu.cn (S.W.); jimjiang09@163.com (S.J.); zhaog@advaccine.com (G.Z.); 2 Shanghai Institute of Infectious Disease and Biosecurity, Fudan University, Shanghai 200032, China

**Keywords:** tumor-cell-based vaccine, inactivated tumor cells, antitumor growth, regulatory T cells, anti-CD25 antibody

## Abstract

Cancer is a major public health threat, and researchers are constantly looking for new ways to develop effective treatments. One approach is the use of cancer vaccines, which work by boosting the body’s immune system to fight cancer. The goal of this study was to develop an effective cancer vaccine using rendered-inactive tumor cells. A CMS5 fibrosarcoma tumor model in BALB/c mice and an E.G7 lymphoma tumor model in C57BL/6 mice were used to evaluate how mitomycin C-inactivated tumor cells mediated tumor protection. The results showed that immunization with inactivated CMS5 cells significantly improved tumor suppression after a challenge with live CMS5 tumor cells, but no effect was observed using the E.G7 tumor model. The results suggested that DC (dendritic cell) responses to tumor antigens are critical. The maturation and activation of DCs were effectively promoted by mitomycin C-treated CMS5 cells, as well as enhanced phagocytosis ability in vitro. The tumor-protective effects established by the vaccination of inactivated CMS5 cells were CD8+ T cell-dependent, as the antitumor responses disappeared after eliminating CD8+ T cells. It was found that the tumor-prevention efficacy was dramatically increased by combining inactivated CM55 tumor cells with anti-CD25 antibodies to temporarily deplete Treg cells (regulatory T cells). This strategy could also significantly induce the rejection against E.G7 tumors. In addition, vaccination with anti-CD25 antibodies plus inactivated CMS5 cells elicited antitumor responses against heterologous tumors. According to the findings of this study, combining the immunization of inactivated tumor cells with an anti-CD25 antibody may be an effective method for cancer prevention.

## 1. Introduction

Tumor-cell-based vaccines have long been developed since 1959, when the first cancer vaccine study with an adjuvanted tumor lysate was published [1], and can be prepared using irradiated whole tumor cells or tumor cell lysates from both autologous and allogenic tumors [2]. As tumor-cell-based vaccines include pan-spectra tumor antigens, they can potentially induce pan-clone-specific T cells and broaden the immune response [3]. Although tumor-cell-based vaccines contain a wide range of antigens, tumor cells can still evade immunosurveillance through genetic mutation, leading to the presence of the most dangerous tumor cells with neoantigens. While cancer vaccines have shown promise in preclinical studies, their effectiveness in clinical trials has been limited by various factors, such as insufficient tumor immunogenicity and immunosuppression in a host. Regulatory T cells (Tregs) are an important T cell subpopulation that is critical for regulating the homeostasis of the immune system [4]. Tregs were found to be enriched in a tumor environment and suppress antitumor immunity [5,6]. Therefore, Tregs have been studied as a therapeutic target for cancer therapy [7]. CD25 (IL-2 receptor a-chain) is highly expressed on Tregs and has been demonstrated to be a selective target for Treg depletion [8]. Further research is needed to overcome these challenges and develop more effective cancer vaccines [9,10,11,12]. Tumor-cell-based vaccines can be used in conjunction with approved immunotherapies or antibodies against Tregs to improve their immunogenicity and inhibit immunosuppression [13,14,15,16].

In this study, we investigated the immune protective effect of a vaccination strategy using mitomycin C (MMC)-treated tumor cells in combination with an anti-CD25 antibody in mouse models. We found that this vaccination strategy induced broad-spectrum protection against homogeneous and heterologous tumor cells.

## 2. Materials and Methods

### 2.1. Mice

BALB/c mice and C57BL/6 (6–8 weeks of age, female) were obtained from Shanghai JieSiJie Laboratory Animal Co., Ltd. All of the mice were housed under pathogen-free conditions. The animal experiments were performed according to the guidelines approved by the Experimental Animal Ethics Committee of Shanghai Medical College (reference number: 20160225-115). All of the experiments were carried out in compliance with relevant guidelines and regulations, including the Animal Research: Reporting of In Vivo Experiments (ARRIVE) guidelines.

### 2.2. Tumor Cell Lines

CMS5 tumor cells, E.G7 tumor cells, and 4T1 tumor cells were gifted by Dr. Zhenzhou Wu (NanKai University, Tianjin, China), Dr. Minghui Zhang (Tsinghua University, Beijing, China), and Dr. Xunbin Wei (Shanghai Jiao Tong University, Shanghai, China), respectively. CT-26 tumor cells were purchased from ATCC (CRL-2638). All tumor cells were cultured in RPMI 1640 (BI) supplemented with 10% fetal bovine serum (BI), 100 U/mL penicillin, and 100 mg/mL streptomycin. Cells were passaged via the use of 0.25% trypsin-EDTA (Gibco).

### 2.3. Inactivated Tumor Cells’ Preparation and Immunization

CMS5 cells or E.G7 cells were incubated with 60 μM of mitomycin C (Sigma, Kanagawa, Japan) in PBS for 30 min [17]. CMS5 cells were adjusted to 3 × 10^7^/mL, while E.G7 cells were to 1 × 10^7^/mL. Inactivated tumor cells at 100 μL per mouse were injected subcutaneously (s.c.).

### 2.4. Tumor Cell Challenge

Mice were challenged with CMS5 cells at 3 × 10^7^/mL, E.G7 cells at 1 × 10^7^/mL, 4T-1 cells at 5 × 10^6^/mL, or CT-26 cells at 1 × 10^7^/mL, 100 μL in PBS. Tumor development was monitored every two days with a caliper. The tumor area was calculated as the tumor length × width. Mice were terminated through CO_2_ euthanasia followed by cervical dislocation when the tumor diameter reached 20 mm.

### 2.5. Antibody Injection

Anti-mouse CD25 antibodies (clone PC61, BioXcell, Lebanon, NH, USA) were injected at an amount of 200 μg per mouse i.p. three days before tumor cell vaccination. Anti-mouse CD8a antibodies (clone YTS 169.4, BioXcell) were injected at an amount of 200 μg per mouse i.p. once a week from one day before the tumor cell challenge.

### 2.6. Assessment of Apoptosis

To analyze apoptosis, cells were treated with 60 μM of mitomycin C for 30 min. Cell death was assessed after 48 h via the use of an Annexin V and 7-aminoactinomycin (7-AAD) apoptosis kit (88–8005-74; eBioscience, San Diego, CA, USA). Samples were acquired with LSRFortessa (BD Biosciences, Franklin Lakes, NJ, USA) and analyzed with FlowJo software.

### 2.7. Dendritic Cell Culture

The mice were sacrificed, and long bones (thigh bone and shin bone) were separated to collect bone marrow cells. Cells were then cultured with a medium containing 20 ng/mL of granulocyte-macrophage colony-stimulating factor (GM-CSF) and 5 ng/mL of IL-4 (PeproTech, Cranbury, NJ, USA). To remove nonadherent cells, the medium was changed every two days. The BMDCs (bone marrow-derived dendritic cells) were harvested for further analysis on day 5 [18].

### 2.8. Immunogenic Cell Death Measurements in Cancer Cell Lines

Cells were seeded into 6-well plates (3 × 10^5^ cells per well) overnight and then treated with 60 μM of mitomycin C for 30 min. Cell death was analyzed 48 h later via flow cytometry for calreticulin (ADI-SPA-601PE-F, enzo life sciences) expression, and supernatants were used to detect HMGB1 via an ELISA (6010, Chondrex, Woodinville, WA, USA), respectively.

Phagocytosis was performed by coculturing inactivated tumor cells with BMDCs. Tumor cells were stained with 2.5 μM of CFSE dye (C34573, Invitrogen, Waltham, MA, USA), and seeded in 48-well plates. BMDCs were stained with 1 μM of efluor 450 (65–0842-85, eBioscience). BMDCs were cocultured with tumor cells in a ratio of 1:1 or 1:3 and incubated for 2 h at 37 °C. BMDCs were collected for the flow cytometry analysis of phagocytosis.

BMDC maturation was assessed after coculturing with MMC-treated or untreated tumor cells. After 24 h, BMDCs were stained with anti-mouse CD11c and anti-mouse CD80 (BD Biosciences), and then analyzed via flow cytometry.

## 3. Results

### 3.1. Tumor Protection Achieved by Vaccination with Inactivated Tumor Cells

**Figure 1:**
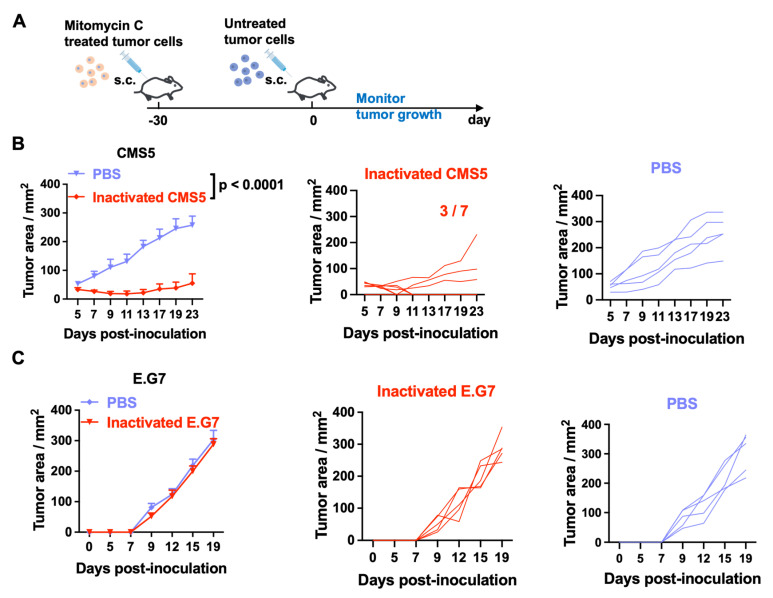
Tumor protection achieved by vaccination with inactivated tumor cells. (**A**) Schematic illustration of mitomycin C (MMC)-treated tumor cells and tumor challenge. Tumor cells were incubated with MMC for 30 min as the inactivated tumor vaccine, which were injected s.c. into the right flank of mice. Mice were challenged with live tumor cells after 30 days (inactivated CMS5/ E.G7 group). Mice injected with PBS at −30 days and then challenged with live tumor cells were set as the control group (PBS). Tumor development was monitored. (**B**) Tumor development in the CMS5 tumor model. (**C**) Tumor development in the E.G7 tumor model.

To investigate the effect of inactivated tumor cell vaccination on immune protection, fibrosarcoma CMS5 of BALB/c mice and lymphoma E.G7 of C57BL/6 mice were used and treated with mitomycin C (MMC) in vitro. MMC is a DNA crosslinker that is commonly used in cancer treatment as a chemotherapy medication. At −30 days, MMC-treated CMS5 cells or E.G7 cells were injected s.c. into the right flank of BALB/c or C57BL/6 mice. Mice were challenged with homogeneous live tumor cells after 30 days (Figure 1A). The control group received PBS at −30 days before being challenged with live tumor cells for day one. Tumor developments were monitored for all of the groups. CMS5 tumors in the control group grew aggressively, as shown in Figure 1B. In contrast, in mice vaccinated with inactivated CMS5 cells, tumor growth was significantly delayed. Moreover, three of the seven mice showed no solid tumor formation. No significant difference in tumor growth was observed between the PBS and E.G7 tumor cell vaccination groups in the E.G7 tumor model (Figure 1C). These results suggest that inactivated tumor cell vaccination protected against a homogenous tumor cell challenge in the CMS5 tumor model but had no effect in the E.G7 tumor model.

### 3.2. The Levels of Danger Signals and DC Maturation Were Correlated with the Type of Tumor Cells Treated by MMC

**Figure 2:**
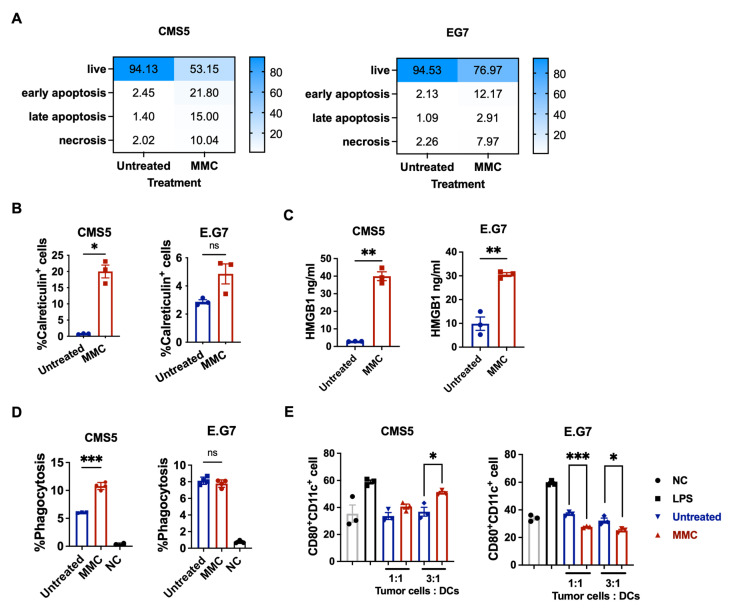
The levels of danger signals and DC maturation were correlated with the type of tumor cells treated by MMC. (**A**) Tumor cell apoptosis and necrosis. (**B**) Calreticulin translocation. Tumor cells were incubated with MMC for 30 min. The calreticulin translocation was detected via flow after 48 h. (**C**) HMGB1 secretion. Tumor cells were incubated with MMC for 30 min. HMGB1 secretion to the medium was detected via an HMGB1 ELISA kit after 48 h. (**D**) DC phagocytosis of tumor cells. Tumor cells were incubated with MMC for 30 min. Tumor cells were cocultured with BMDCs for 6 h. The phagocytosis of BMDCs was detected via FCM. (**E**) DC maturation. Tumor cells were incubated with MMC for 30 min. Tumor cells were cocultured with BMDCs for 24 h. The expression of CD80 in BMDCs were detected via FCM. Two-way ANOVA and one-way ANOVA tests were used to determine statistical significance. * *p* < 0.05, ** *p* < 0.01, *** *p* < 0.001. ns: not significant.

To explore the discrepancies between the two tumor cells, the cell changes induced through MMC treatment were investigated, including cell apoptosis, calreticulin translocation, and high-mobility group box 1 (HMGB1) secretion, to determine why inactivated CMS5 and E.G7 cells conferred distinct tumor protection effects. Following CMS5 or E.G7 treatment with 60 μM of MMC at 37 °C for 30 min, the cells’ apoptosis, calreticulin translocation, and HMGB1 secretion were detected after 48 h. CMS5 had a higher apoptosis and calreticulin positive ratio than E.G7, according to flow cytometry data (Figure 2A,B). CMS5 cells treated with 60 μM of MMC secreted a higher level of HMGB1 compared with E.G7 cells after collecting their culture medium and analyzing it with a HMGB1 ELISA kit (Figure 2C). Based on these results, it was demonstrated that CMS5 treated with MMC induced more immunogenic cell death. As these damage-associated molecular patterns (DAMPs) could promote the uptake of dying tumor cells via a phagocytosis mechanism and the subsequent activation of dendritic cells (DCs), we employed a flow cytometry analysis to assess DC phagocytosis of tumor cells and tumor cell stimulation of DC maturation. The percentages of CMS5 cells phagocytized by BMDCs were significantly increased with MMC treatment. But no such difference was observed with E.G7 cells (Figure 2D). Furthermore, MMC treated CMS5 cells induced elevated DCs expression of CD80, but MMC-treated E.G7 stimulated less CD80 expression on DCs in comparison with untreated E.G7 (Figure 2E). These results indicate that CMS5 treatment with MMC induces the immunogenic cell death and activation of dendritic cells (DCs), which may process and present tumor antigens to T cells, resulting in a robust anticancer immune response. In addition, these results may explain why the E.G7 model failed to produce a significant antitumor response.

### 3.3. Tumor Rejection after Vaccination with Inactivated CMS5 Tumor Cells Was Mediated by CD8+ T Cells

**Figure 3:**
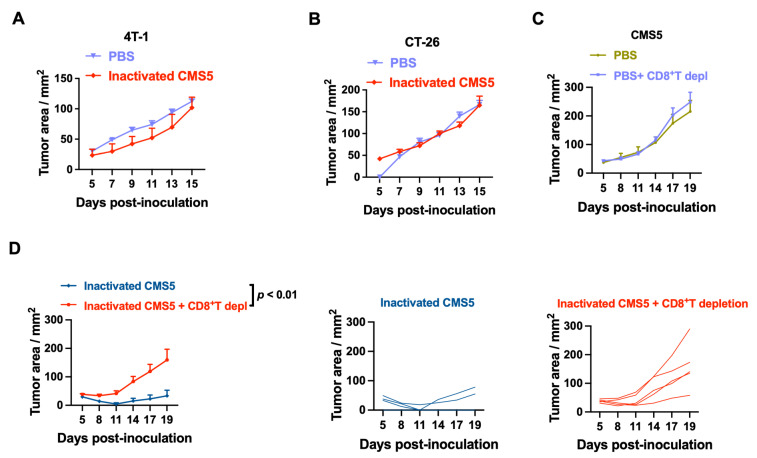
Tumor rejection after vaccination with inactivated CMS5 tumor cells was mediated by CD8+ T cells. After 30 days following immunization with inactivated CMS5 tumor cells, mice were challenged with heterologous tumor cells, 4T-1 or CT-26. Mice injected with PBS and challenged with 4T-1 or CT-26 tumors served as the control. Tumor progression was monitored. (**A**) 4T-1 tumor growth after mice vaccinated with inactivated CMS5 tumor cells or PBS. (**B**) CT-26 tumor growth after mice vaccinated with inactivated CMS5 tumor cells or PBS. CD8+ T cells were depleted by injecting i.p. 200 μg of anti-CD8 antibodies one day before the CMS5 tumor challenge. Tumor growth was monitored. (**C**) CMS5 tumor growth. (**D**) Tumor growth after mice were vaccinated with inactivated CMS5 tumor cells.

To investigate if the tumor protection was tumor-cell-specific, mice vaccinated with inactivated CMS5 cells were challenged with either 4T-1 or CT-26 tumor cells. As shown in Figure 3A,B, there was neither protection against the two heterologous tumor cell challenges nor a significant difference compared to the PBS group. This result suggests that an inactivated CMS5 vaccine cannot protect against heterozygous tumors using this protocol.

To determine whether inactivated-tumor-cell-vaccination-induced tumor protection was CD8+ T cell-dependent, anti-CD8 antibodies were used to deplete CD8+ T cells during a CMS5 tumor challenge. Beginning one day prior to the CMS5 tumor challenge, both the PBS-treated and inactivated-CMS5-vaccinated groups received once-weekly anti-CD8 antibody treatments. In the PBS group, CD8+ T cell depletion had no effect on CMS5 tumor growth (Figure 3C), whereas CD8+ T cell depletion in the CMS5 vaccination group reversed the suppression of tumor effect (Figure 3D). This result demonstrates that CD8+ T cells mediate the protection against tumors induced by vaccination against CMS5 tumors.

### 3.4. Combining Inactivated Tumor Cells with Anti-CD25 Antibodies Enhanced the Antitumor Effect on Tumor Challenge

**Figure 4:**
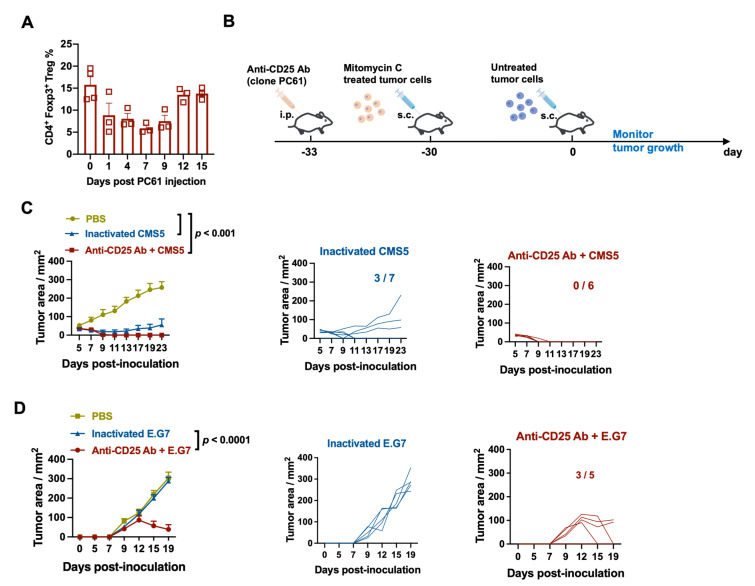
Combining inactivated tumor cells with anti-CD25 antibodies enhanced the antitumor effect to tumor challenge. (**A**) Schematic illustration of inactivated tumor cells combined with anti-CD25-antibody-promoted tumor protection. Mice were injected i.p. with 200 μg of anti-CD25 antibodies (clone PC61) at −33 days. Then, MMC-treated inactivated tumor cells were vaccinated at −30 days. The mice were then challenged with untreated tumor cells. Tumor growth was monitored. (**B**) The effect of anti-CD25-antibody-mediated Treg depletion. The level of CD4+Foxp3+ Treg cells in PBMCs were analyzed through flow cytometry. (**C**) Inactivated tumor cells combined with anti-CD25 antibody vaccination induced tumor protection in the CMS5 tumor model. (**D**) Inactivated tumor cells combined with anti-CD25 antibody vaccination induced tumor protection in the E.G7 tumor model.

It is known that Treg cells inhibit antitumor immune responses and desensitize DC presentation. Anti-CD25 antibodies (PC61) are monoclonal antibodies that target CD25 on the surface of Treg cells and are commonly used to deplete Treg cell function. To examine the effect of PC61 on Tregs in peripheral blood, a single i.p. administration of PC61 was administered. The percentage of Treg cells decreased to less than 5% from the normal level of 15% of CD4+T cells on day 1, and remained less than 5% until day 9. On day 12, the number of Treg cells returned to normal levels (Figure 4A). The antitumor procedure incorporated this temporary and partial depletion of Treg cells. Three days prior to the immunization with inactivated tumor cells, as shown in Figure 4B, PC61 was administered i.p., followed by a challenge with live tumor cells. Using flow cytometry, the effect of the anti-CD25 antibody on CD4+Foxp3+ Treg cells in peripheral blood mononuclear cells (PBMCs) was determined. Following the introduction of homologous tumor cells, the proliferation of the tumor was observed. In the CMS5 tumor model, the PBS-injected group displayed progressive tumor growth. In the inactivated CMS5 vaccine group, three out of seven mice were tumor-free, whereas in the inactivated CMS5 vaccine plus anti-CD25 antibody treatment group, all six animals were tumor-free (Figure 4C). Three out of five mice were tumor-free following the E.G7 tumor cell challenge when an inactivated E.G7 tumor vaccine was combined with a single PC61 treatment, whereas tumors developed aggressively in the PBS and inactivated E.G7 vaccine groups (Figure 4D). These findings suggest that the anti-CD25-antibody-mediated transient inhibition of Treg cells could significantly boost the antitumor responses induced by the inactivated tumor vaccine.

### 3.5. Anti-CD25 Antibody Combined with Inactivated-CMS5-Cell-Vaccination Induced Antitumor Responses against Heterologous Tumor Challenge

**Figure 5:**
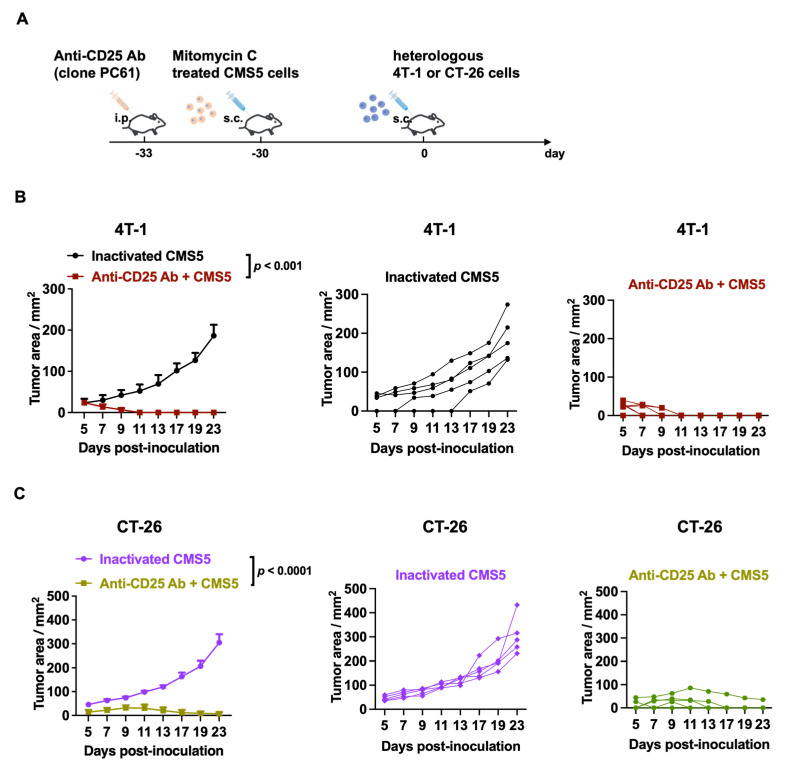
Anti-CD25 antibodies combined with inactivated CMS5 cell vaccination induced antitumor responses against heterologous tumor challenges. (**A**) Schematic illustration of heterologous tumor challenge to mice vaccinated with anti-CD25 antibodies plus inactivated CMS5 tumors. (**B**) Tumor development in mice vaccinated with inactivated CMS5 cells or anti-CD25 antibodies plus inactivated CMS5 cells and then challenged with 4T-1 tumors. (**C**) Tumor development in mice vaccinated with inactivated CMS5 cells or anti-CD25 antibodies plus inactivated CMS5 cells and then challenged with CT-26 tumors.

To investigate the responses of vaccinated mice to heterologous tumors, anti-CD25-AB- plus inactivated-CMS5-vaccinated mice were challenged with heterologous tumor cells (4T-1 or CT-26), and tumor development was monitored (Figure 5A). Figure 3A depicts the aggressive progression of 4T-1 tumors in the inactivated-CMS5-vaccinated cohort. In stark contrast, vaccination with anti-CD25 antibodies and CMS5 cells eradicated 4T-1 tumors (Figure 5B). CT-26 tumors grew swiftly in the inactivated CMS5 vaccine group for the CT-26 challenge investigation, as depicted in Figure 3C. In contrast, the anti-CD25 antibody and inactivated CMS5 vaccination group significantly inhibited CT-26 tumor growth (Figure 5C). These results indicate that vaccination with anti-CD25 antibodies together with the inactivated CMS5 vaccine induced antitumor responses against heterologous 4T-1 and CT-26 tumors, suggesting that the addition of anti-CD25 antibodies yielded a broader spectrum of protection.

## 4. Discussion

The objective of this study was to create an effective cancer vaccine based on inactive tumor cells. Using mitomycin C-inactivated tumor cells, a CMS5 fibrosarcoma tumor model in BALB/c mice and an E.G7 lymphoma tumor model in C57BL/6 mice were investigated. It was demonstrated that vaccination with an inactivated-CMS5-cell-based vaccine can significantly enhance tumor suppression following a challenge with live CMS5 tumor cells, whereas the E.G7 model failed to show such an effect. In addition, it was found that MMC-sensitive tumor cells are crucial to the efficacy of the treatment, possibly because they induce DC activation and tumor antigen presentation. The tumor-protective effects were dependent on CD8+ T cells, as tumor cells can resume development once CD8 T is depleted. Furthermore, it was demonstrated that the combination of anti-CD25 antibodies, which temporarily deplete Treg cells, with an inactivated CM55 tumor vaccine can substantially enhance the antitumor effect against homologous and heterologous tumor challenges.

Mitomycin C (MMC) is an alkylating agent with an antiproliferative activity that inhibits the DNA synthesis of cells exhibiting the highest rate of mitosis. It is an anticancer antibiotic that is widely used in clinical chemotherapy. MMC was reported to promote immunogenic cell death. In this process, the dying cells release DAMPs and promote the maturation of DCs as well as the activation of cytotoxic T cells [19,20]. During the initiation stage of MMC-induced immunogenic cell death of tumor cells, chaperone calreticulin translocates to the surface of membrane, which represents an immunogenic “eat-me” signal for DCs in phagocytosis [21]. At the late stage of apoptosis, high-mobility group box 1 (HMGB1) protein is passively released for binding to Toll-like receptor 4 on DCs to promote antigen presentation [22]. CMS5 tumor cells are more sensitive to the MMC treatment, as evidenced by a higher proportion of calreticulin and HMGB1-positive cells, compared to E.G7 tumor cells (Figure 2), which are more likely to undergo immunogenic cell death. The vaccination of inactivated CMS5 cells treated with MMC-induced tumor rejection is dependent not only on MMC’s cytotoxic potential but also on its ability to exert immunostimulatory activities by inducing immunogenic cell death. 

Phagocytosis is an important process for DCs during the antitumor response. Because it allows them to engulf and process tumor antigens, which can then be presented and cross-presented to T cells to activate an immune response [23,24]. Inactivated CMS5 cells exhibited a robust ability to promote DCs’ phagocytosis of tumor antigens and DC maturation, resulting in a more potent antitumor response, as observed in Figure 1.

Our group and other recent studies have demonstrated that tumor growth was completely inhibited after the depletion of Treg cells within the tumor-side draining lymph node [25,26]. Therefore, targeting Treg cells represents a promising approach in tumor therapy. The depletion of Treg cells during tumor growth can have a significant impact on the immune response to cancer [27]. Combining the immunization of inactivated tumor cells with an anti-CD25 antibody may be an effective method of cancer prevention [28]. The removal of Treg cells using cell-depleting anti-CD25 antibodies effectively eradicated a variety of inoculated syngeneic tumors [29,30]. In a murine melanoma model, the application of anti-CD25 monoclonal antibodies was also found to improve dendritic cell–tumor fusion vaccine efficacy [31]. Anti-CD25 antibodies have shown the potential of CD25+ T cell depletion with an enhanced antitumor immune response when combined with vaccine immunotherapies [32]. However, previous anti-CD25 monoclonal antibodies have had limited efficacy in tumor inhibition and particularly failed clinically, possibly because CD8+ T cells with a high expression of CD25 were also depleted [8,33]. In this study, it is surprising to us that anti-CD25 antibodies can robustly enhance efficacy against both homogeneous and heterologous tumor challenges (Figure 5). This may also due to enhanced DC activation and high effective tumor antigen cross-presentations, since the PC61 can block early tumor establishment by depleting CD25+ Tregs and inducing CD8 T cell-mediated systemic antitumor immunity [30]. Therefore, the use of anti-CD25 antibodies to deplete CD25+ T cells has shown promising results in enhancing efficacy against both homogeneous and heterologous tumor challenges. Overall, the study demonstrates that a treatment combining the immunization of inactivated tumor cells with an anti-CD25 monoclonal antibody is an effective method against cancer growth; however, further research is needed to determine the efficacy and safety of this approach in humans.

The specificity of tumor targeting is enhanced by the emergence of neoantigens, which arise due to the occurrence of tumor antigen mutations throughout the process of carcinogenesis and tumorigenesis. Although the efficacy of this “catching up gaming technique” has been shown to have limited success in recent years, the use of neoantigen strategies presents some technical obstacles and limitations. The process of cancer development is a multifaceted and dynamic evolutionary occurrence marked by genetic instability, which is further amplified under the surveillance of a host immune system. The use of algorithms in the identification of neoantigens has faced many challenges in terms of prediction parameters and prioritization settings [34]. Existing algorithms may miss undetected or unexperienced antigens. In addition, it should be noted that neoantigen-based vaccines have a limited ability to target tumor antigens, and relying solely on neoantigen vaccines is inadequate for the eradication of rapidly evolving cancers [35]. In contrast, our study indicated that killed tumor cells as a vaccine, if taken from cancer patients, may include all types of antigens, such as tumor-specific antigens (TSAs), neo-antigens, and hidden antigens. These antigens were capable of being exposed to the immune system of a host, enabling the host’s immune mechanisms to recognize and target them. The findings of our study demonstrate that the application of inactivated tumor cells and anti-CD25 antibodies led to the elicitation of extensive antitumor reactions, successfully protecting against both homologous and heterologous tumor challenges.

## 5. Conclusions

While the study presents promising results in the use of immunization and anti-CD25 monoclonal antibodies in cancer treatment/prevention, more extensive research and clinical trials are necessary to assess the safety and efficacy of this approach in humans. Further studies can shed light on the potential of this combination therapy as a viable option for cancer treatment/prevention.

## Data Availability

The datasets generated during and/or analyzed during the current study are available from the corresponding author upon reasonable request.
